# Healthcare expenditure on Indigenous and non-Indigenous Australians at high risk of cardiovascular disease

**DOI:** 10.1186/s12939-017-0610-2

**Published:** 2017-06-23

**Authors:** Blake Angell, Tracey-Lea Laba, Tom Lung, Alex Brown, Sandra Eades, Tim Usherwood, David Peiris, Laurent Billot, Graham Hillis, Ruth Webster, Andrew Tonkin, Christopher Reid, Barbara Molanus, Natasha Rafter, Alan Cass, Anushka Patel, Stephen Jan

**Affiliations:** 1 The Poche Centre for Indigenous Health, University of Sydney, Sydney, NSW Australia; 2 The George Institute for Global Health, UNSW Sydney, Level 10 King George V Building, 83-117 Missenden Road, Camperdown, 2050 NSW Australia; 3 Sydney Medical School, University of Sydney, Sydney, Australia; 4 Baker IDI, Melbourne, VIC Australia; 5 South Australia Health and Medical Research Institute, Adelaide, Australia; 6 WA Health, Perth, Australia; 7 Cardiovascular Research Unit, Department of Epidemiology and Preventive Medicine, Monash University, Melbourne, Australia; 8 School of Public Health, Curtin University, Perth, Australia; 9 Royal College of Surgeons in Ireland, Dublin, Ireland; 10 Menzies School of Health Research, Darwin, Australia

**Keywords:** Healthcare expenditure, Australia, Indigenous health, Chronic disease, Cardiovascular disease

## Abstract

**Background:**

In spite of bearing a heavier burden of death, disease and disability, there is mixed evidence as to whether Indigenous Australians utilise more or less healthcare services than other Australians given their elevated risk level. This study analyses the Medicare expenditure and its predictors in a cohort of Indigenous and non-Indigenous Australians at high risk of cardiovascular disease.

**Methods:**

The healthcare expenditure of participants of the *Kanyini Guidelines Adherence with the Polypill (GAP)* pragmatic randomised controlled trial was modelled using linear regression methods. 535 adult (48% Indigenous) participants at high risk of cardiovascular disease (CVD) were recruited through 33 primary healthcare services (including 12 Aboriginal Medical Services) across Australia.

**Results:**

There was no significant difference in the expenditure of Indigenous and non-Indigenous participants in non-remote areas following adjustment for individual characteristics. Indigenous individuals living in remote areas had lower MBS expenditure ($932 per year *P* < 0.001) than other individuals. MBS expenditure was found to increase with being aged over 65 years ($128, *p* = 0.013), being female ($472, *p* = 0.003), lower baseline reported quality of life ($102 per 0.1 decrement of utility *p* = 0.004) and a history of diabetes ($324, *p* = 0.001), gout ($631, *p* = 0.022), chronic obstructive pulmonary disease ($469, *p* = 0.019) and established CVD whether receiving guideline-recommended treatment prior to the trial ($452, *p* = 0.005) or not ($483, *p* = 0.04). When controlling for all other characteristics, morbidly obese patients had lower MBS expenditure than other individuals (−$887, *p* = 0.002).

**Conclusion:**

The findings suggest that for the majority of participants, once individuals are engaged with a primary care provider, factors other than whether they are Indigenous determine the level of Medicare expenditure for each person.

**Trial registration:**

Australian New Zealand Clinical Trials Registry ACTRN 126080005833347.

## Background

The burden of chronic diseases facing Australians has grown significantly over recent decades making providing appropriate care for these patients increasingly complex [1, 2]. Studies have shown a growing burden on health system expenditure in Australia as a result of the increased prevalence of obesity and diabetes for example [3], while other chronic diseases such as chronic obstructive pulmonary disease (COPD), gout and cardiovascular diseases (CVD) are likely to place significant demands on health system resources.

Greater chronic disease multi-morbidity is an important contributor to the health gap between Aboriginal and Torres Strait Islander (Indigenous) Australians and non-Indigenous Australians [4]. Ensuring that Indigenous Australians have access to effective healthcare services is a central component of government attempts to eradicate these inequalities [5]. Nonetheless, a number of barriers prevent Indigenous Australians from accessing appropriate health services including financial, cultural, geographic and health-literacy impediments [6, 7].

### Healthcare service use of indigenous Australians

Without controlling for the relative need of the two groups, the average public spend on healthcare for an Indigenous Australian is estimated at $1.47 for every $1 spent for the care of a non-Indigenous Australian [8]. The distribution of this spend differs markedly between the two groups, however, with Indigenous Australians utilising significantly less Medicare Benefit Schedule (MBS) and medication expenditure (at a rate of 0.63 and 0.44 respectively) than other Australians but having much higher average hospital expenditure (1.76 times higher than non-Indigenous Australians). MBS expenditure incorporates general practitioner and specialist visits and diagnostic tests. Given the substantial disparities in health outcomes facing Indigenous Australians, many have questioned whether the expenditure on Indigenous individuals, in particular for primary healthcare, should be higher relative to non-Indigenous Australians [6, 9]. Limited research has been able to effectively adjust for the different clinical risk profiles of the two populations. One study that was able to do so (through controlling for self-reported indicators of ill health) found that Indigenous Australians used more healthcare services than other Australians [10].

This study investigates these issues using patient-level clinical data to examine the relative patterns of healthcare expenditure of Indigenous and non-Indigenous individuals at high-risk of CVD and investigate the predictors of expenditure across the two groups. Understanding the healthcare utilisation patterns of these populations is important to ensure that the health system is providing the level of care required to close the gap in health outcomes between Indigenous and non-Indigenous Australians and appropriate levels of care to those living with chronic diseases.

## Methods

### Participants

Data were collected during the *Kanyini Guidelines Adherence with the Polypill (GAP)* randomised controlled trial testing the effectiveness of a cardiovascular combination polypill (containing aspirin, simvastatin and two low dose blood pressure lowering agents) in Indigenous and non-Indigenous adults over 18 years at high-risk of CVD. High-risk of CVD was defined as either an established diagnosis of CVD or an estimated five-year risk of CVD greater than 15 percent based on the Framingham risk equation (with an additional five percent increment for Indigenous participants) [11]. Participants were excluded if it was deemed clinically inappropriate to alter their medications. Participants had a median follow-up of 19 months (maximum follow up of 36 months). Further details of the trial design and randomisation process are presented elsewhere [11]. The trial was approved by human research ethics committees in all relevant jurisdictions.

### Service expenditure

Data collected included data from the Medicare Benefits Schedule (MBS), which records government and patient out of pocket expenditure (government legislated co-payments) on general practitioner and specialist visits and diagnostic tests, linked with data from the clinical trial [12]. MBS data are automatically collected at the point of service when the healthcare provider bills the government or patient for the service rendered and participants consent for these data to be linked to the data collected through the trial. Pharmaceutical Benefit Schedule (PBS) data were also collected for the trial, however, this did not include: (1) the polypill treatment used in the trial (as it was not PBS-approved); (2) pharmaceutical treatments falling under the general co-payment threshold; and (3) those medicines accessed by people of remote Aboriginal Health Services under the provisions of section 100 of the National Health Act (1953) [13]. As such, the average MBS expenditure per patient per follow-up year was used as the outcome variable for the analysis.

### Geographic setting

The trial was conducted within Indigenous-specific (12 centres) and other primary-care providers (21 centres) in urban, rural and remote settings around Australia. Urban, regional or remote areas were classified based on definitions of the Australian Bureau of Statistics [14]. For this analysis, ‘remote’ is used to refer to those living in either remote or very remote areas under these definitions.

### Statistical analysis

The average annual MBS benefit of the two groups was modelled using linear regression methods using Stata 13.1 (StataCorp). To account for the skewness of the data, the outcome variable was log-transformed and then estimated using linear regression models [15]. The adjusted log means were then transformed back to a dollar scale using a smearing estimate in order to derive unbiased estimates of mean costs [16]. Medicare benefit expenditure was estimated within key socioeconomic and demographic variables collected from study participants at the baseline visit including gender, income, whether they received the polypill or usual care, Indigenous status, remoteness, highest education level attained, the presence of certain chronic diseases (diabetes, COPD, gout and morbid obesity defined as having a body mass index (BMI) greater than 40), whether a person was a primary or secondary prevention patient and whether they were on appropriate medication prior to entering the trial with and without covariate adjustments. The quality of life of the patient measured at baseline using the EQ-5D and converted to a summary score using Australian based estimates was included as an explanatory variable in the analysis [17]. Non-significant baseline covariates (*P* > 0.1) were removed via backwards stepwise elimination. Age of participants and morbid obesity were included as dummy variables in the analysis due to an observed differential impact for those aged 65 years and over and those with a BMI over 40. Indigenous status was captured through two dummy variables, one indicating if they lived in a remote area and another if they lived in an urban or regional setting. This was necessary as the impact of living in a rural area could not be isolated for non-Indigenous Australians as there were too few non-Indigenous people in the sample living in rural or remote locations. All costs are presented in 2012 Australian dollars.

## Results

### General characteristics

Complete data were available for 535 participants in the *Kanyini GAP* study which represented 88% of the total trial population^1^. Table 1 summarises the general characteristics of participants and shows the average MBS expenditure by each category shown. It is important to highlight that the individual characteristics vary across the urban and regional Indigenous, Remote Indigenous and non-Indigenous groups and as such, this average expenditure should be interpreted with caution. The sample included 48% Indigenous people, had an average age of 63 years and 36% were female. Overall, 67% of participants were from urban areas, 12% from regional areas and 21% were from remote areas. Thirty-eight percent of the patient cohort were receiving treatment for the primary prevention of CVD and 62% had an established diagnosis of CVD prior to entering the study. The average BMI of participants was 31 with 9.7% (52 participants) of the cohort classified as morbidly obese. The average five -year cardiovascular risk score of primary prevention patients was 18.7%, and this was similar for Indigenous (18.7%) and non-Indigenous (18.6%) individuals. On average, Indigenous participants were younger, more likely to be receiving primary rather than secondary prevention care and more likely to live in rural or remote locations than their non-Indigenous counterparts.

**Table 1:** General Characteristics of Cohort

	Total		Urban and Regional Indigenous		Remote Indigenous		Non-Indigenous	
	*n(%)*	*Average MBS Expenditure*	*n(%)*	*Average MBS Expenditure*	*n(%)*	*Average MBS Expenditure*	*n(%)*	*Average MBS Expenditure*
*Participants*	535^a^	$1,699	146 (27%)	$1,863	113 (21%)	$1,161	276 (52%)	$1,833
*Age, mean (SD)*	63 (12.5)	NA	57 (8.8)	NA	55 (13.6)	NA	70 (9.5)	NA
*Morbidly Obese*	52 (10%)	$1,310	27 (18%)	$1,519	9 (8%)	$637	16 (7%)	$1,336
*Female*	193 (36.1%)	$1,833	56 (39%)	$1,932	49 (43%)	$1,284	88 (32%)	$2,075
Primary Healthcare Provider								
* Aboriginal Medical Service (AMS)*	266 (50%)	$1,580	136 (93%)	$1,901	113 (100%)	$1,161	17 (6%)	$1,805
* Non-AMS*	269 (50%)	$1,816	10 (7%)	$1,346	0 (0%)	NA	259 (94%)	$1,834
Geographical Classification								
* Urban*	356 (67%)	$1,878	102 (70%)	$1,979	0 (0%)	NA	254 (92%)	$1,837
* Regional*	64 (12%)	$1,625	44 (30%)	$1,594	0 (0%)	NA	20 (7%)	$1,693
* Remote and very Remote*	115 (21%)	$1,185	0 (0%)	NA	113 (100%)	$1,161	2 (1%)	$2,565
CVD Risk Group								
* Primary Prevention, not on optimal therapy before the trial*	94 (18%)	$1,421	41 (28%)	$1,623	20 (18%)	$789	33 (12%)	$1,421
* Primary Prevention, on optimal therapy before the trial*	108 (20%)	$1,601	22 (15%)	$1,980	39 (35%)	$959	47 (17%)	$1,957
* Secondary Prevention, not on optimal therapy before the trial*	42 (8%)	$1,941	12 (8%)	$1,819	4 (4%)	$1,346	26 (9%)	$2,089
* Secondary Prevention, on optimal therapy before the trial*	291 (54%)	$1,790	71 (49%)	$1,973	50 (44%)	$1,452	170 (62%)	$1,813
Highest Educational Attainment								
* Primary education or lower*	142 (27%)	$1,513	51 (35%)	$1,703	49 (43%)	$1,041	42 (15%)	$1,834
* Secondary level*	247 (46%)	$1,742	56 (38%)	$1,844	44 (39%)	$1,236	147 (53%)	$1,855
* Post-secondary*	146 (27%)	$1,807	39 (27%)	$2,099	20 (18%)	$1,288	87 (32%)	$1,795
*Risk Score of primary prevention patients, mean (SD)*	18.7 (5.9)	NA	19.2 (5.8)	NA	18.3 (6.8)	NA	18.6 (5.2)	NA
*Presence of COPD at baseline of trial*	45 (8%)	$2,130	23 (16%)	$2,146	5 (4%)	$2,405	17 (6%)	$2,027
*Presence of diabetes at baseline of trial*	289 (54%)	$1,741	98 (67%)	$1,961	83 (73%)	$1,083	108 (39%)	$2,047
*Presence of gout at baseline of trial*	109 (20%)	$2,054	30 (21%)	$2,139	14 (12%)	$1,340	65 (24%)	$2,169
*Received polypill intervention*	272 (51%)	$1,708	69 (47%)	$2,052	59 (52%)	$1,161	144 (52%)	$1,766

^a^88% of Kanyini GAP trial participants

There were no major differences between *Kanyini GAP* participants who were included and excluded from this analysis (88 subjects) in terms of age, primary prevention risk score, average age or regional classification. A higher proportion of trial participants who did not provide consent for linkage to their MBS records were Indigenous (64%).

### Service utilisation

On average, participants accessed a total of 34 MBS services per year during the trial. The average MBS benefit paid per year for care of the patient cohort was $1,699. Indigenous participants had lower MBS service utilisation across the trial (see Table 2). Remote Indigenous participants had accessed almost 27 MBS services as opposed to 36 for other participants (Indigenous and non-Indigenous) and had an average MBS Benefit expenditure of $1,161 compared to $1,843.

**Table 2:** Average Selected services per follow-up year

	Total	Urban and Regional Indigenous	Remote Indigenous	Non-Indigenous
Number of MBS Services	34	36	27	36
Average Total MBS Benefit Expenditure per year	$1,699	$1,863	$1,161	$1,833
Average MBS Out of Pocket Expenditure	$67	$16	$4	$121

### Predictors of service expenditure

Table 3 outlines the predicted variation in MBS benefits based on the model developed. Indigenous patients (living in non-remote areas) were estimated to have an average annual MBS benefit expenditure $108 higher than non-Indigenous participants, however, this difference was not statistically significant (*p* = 0.33). Indigenous people living in remote locations, however, had an average annual MBS benefit expenditure $932 less than other participants (*p* < 0.001). Baseline self-reported quality of life was significantly associated with the MBS benefit expenditure of the patients with expenditure increasing on average $102 for each 0.1 decrement of utility. Morbidly obese patients (BMI > 40) had significantly lower MBS expenditure per year ($887 lower per year, *p* = 0.002). Medicare expenditure was significantly associated with whether a person was a primary or secondary prevention patient and whether they were on appropriate medications before they started the trial: secondary prevention patients who were not on all evidence-based medications prior to the start of the trial were estimated to have service use $483 higher on average than primary prevention patients who were similarly not on appropriate medications (*p* = 0.04). Other socio-demographic variables including the education level and income of the participants were not statistically significant predictors of service use once the other variables were included in the model. The MBS expenditure of participants was not different between the randomisation groups, that is, whether they received the polypill intervention or usual care in the trial.

**Table 3:** Average increases in estimated annual MBS benefit associated with patient characteristics

	Increase in estimated annual MBS Benefit^a^	*p-*Value**
Urban Indigenous	$108	0.326
Baseline quality of life	$102 per 0.1 decrement in utility	0.004
Aged 65 and over	$128	0.013
Female	$472	0.003
Remote Indigenous	-$932	<0.001
Primary prevention and on optimal medications prior to trial	$285	0.193
Secondary prevention and not on optimal medications prior to trial	$483	0.04
Secondary prevention and on optimal medications prior to trial	$452	0.005
Patient history of gout	$631	0.022
Patient history of diabetes	$324	0.001
Patient history of COPD	$469	0.019
BMI > 40	-$887	0.002
Constant	$2,379	<0.001

^a^expenditure increases based on differences in smeared means

**p-values based on natural log-transformed data adjusted for all variables included above

## Discussion

Cardiovascular diseases are estimated to affect 5.2% of Australians [18], 12% of Indigenous Australians [19] and have been estimated to be responsible for approximately a quarter of the gap in health outcomes between Indigenous and non-Indigenous Australians [4, 20]. CVD hospitalisations and death rates in remote and very remote areas of Australia are estimated to be 30% higher than in major cities [21]. Ensuring that Indigenous and remote communities experiencing or at risk of CVD can access appropriate care is vital to closing this gap. This study provides a unique insight into the service use patterns of this high-risk cohort. Average MBS expenditure was lower per capita for Indigenous Australians relative to other participants, in line with national estimates [8]. Most of this difference, however, resulted from remote Indigenous participants receiving significantly lower levels of Medicare-funded care. While this finding is consistent with previous literature highlighting service-gaps and difficulties in providing healthcare services to remote communities, Medicare services are also an incomplete picture of healthcare being received as many services may be funded by other sources including government grants, which previous estimates have suggested could represent up to $1,300 per person per year of services in these areas [22]. This would account for a large part of the difference in Medicare expenditure between remote Indigenous and other participants found in this study. It is worth noting though the potential equity implications resulting from a two-tiered funding system. Most notably, the additional burden on AMS providers who need to apply for and meet other requirements of these grants has been well documented [23, 24].

On the other hand, once demographic and risk-related factors were controlled for there was no significant difference in the MBS expenditure of Indigenous and non-Indigenous participants. For Indigenous individuals living in non-remote locations, other factors were more important in determining their care than their Indigeneity. This suggests that this cohort of high-risk individuals were receiving care equivalent to their non-Indigenous counterparts with similar risk levels. While it could be argued that these individuals should be receiving a greater level of care to ‘close the gap’, this is a promising result particularly given that almost all Indigenous individuals in this cohort received care at an Aboriginal-specific primary healthcare centre. The importance of Aboriginal-specific services and culturally appropriate care has been highlighted repeatedly in the qualitative literature as an essential component in ensuring that appropriate care is received by these communities [25–28]. The findings of this study suggest that in urban areas at least, culturally-specific services can help to overcome the underutilisation of primary care services by Australia’s Indigenous communities. Further it suggests that urban Indigenous specific providers may be overcoming a historical underutilisation of Medicare as a funding source.

We were unable to separate the MBS expenditure into that relating to cardiovascular as opposed to other specialties as the majority of costs were for services such as pathology tests and general consultations which may result from a number of conditions. While a patient history of gout, diabetes and COPD were all associated with significantly higher Medicare expenditure as would be expected, morbidly obese patients were found to be receiving significantly lower levels of care receiving almost $900 less care per year than other participants and an average of under 29 services per year as opposed to 34 for other participants. This goes against findings in the literature that have found increased levels of obesity to be associated with higher expenditure [3] as well as national data indicating that obese people were more likely to see a GP than non-obese individuals [29]. While the reasons underlying this finding are not immediately apparent there are several potential explanations. First, we did not have data on the hospital service use of patients so that the difference in Medicare expenditure may be compensated by hospital expenditure if these morbidly obese individuals used more hospital services. Second, previous studies have not controlled for the presence of multi-morbidities as we have suggesting that perhaps the higher expenditure found in those studies may reflect the presence of other chronic diseases. Third, it may be the case that the morbidly obese patients are either less proactive in seeking out health services or the primary healthcare services may not be meeting the needs of this cohort. Given the increasing prevalence of obesity in Australia, it is vital to ensure that primary care providers effectively meet the healthcare needs of this population to maximise patient outcomes and minimise downstream cost impacts. In any case, this relationship between service use and obesity warrants further investigation.

There were several limitations to this analysis. First, the sample is not representative of the general Australian Indigenous population. Participants were recruited from primary care services such that they were already receiving care and many of the impediments to care discussed in the literature had already been overcome. This might lead to our estimates of expenditure being an overestimate for these communities. Further our focus on Indigenous communities likely means our cohort is not generalizable to the broader Australian population at high-risk of CVD. Second, there were too few non-Indigenous Australians living in remote locations to separate the impact of living in a remote area and that of being Indigenous. While one of the strengths of the paper was our ability to exploit patient-level data to control for risk and other socio-demographic variables there are limitations to the dataset. We did not have access to hospital data and the lack of clarity around potential alternative services being provided particularly in remote areas means that we cannot ascertain the exact level of services being accessed by these communities. Similarly, other characteristics that we could not control for may influence the healthcare spend of these individuals. Finally, our data may be affected by the higher rate of Indigenous participants who did not consent to link their trial and Medicare data.

## Conclusion

Understanding the healthcare utilisation patterns of Indigenous Australians is important to ensure that the health system is providing the level of care required to close the gap in health outcomes between Indigenous and non-Indigenous Australians. This analysis suggests that in an urban setting, once individuals are engaged with a service, culturally-specific care providers can be effective in providing care to this high-risk patient group. Indigenous individuals living in remote areas had significantly lower levels of Medicare funded care, however, we were unable to determine if this was a result of fewer services being received or limitations with our data. Policy efforts to improve access to primary care for Indigenous Australians should focus on getting these communities engaged with care providers and overcoming barriers facing those in remote areas.

## Abbreviations

BMI: Body Mass Index
COPD: chronic obstructive pulmonary disease
CVD: Cardiovascular Diseases
Kanyini GAP: Kanyini Guidelines Adherence with the Polypill pragmatic randomised controlled trial
MBS: Medicare Benefits Schedule
PBS: Pharmaceutical Benefits Schedule

## Footnotes

1 Six hundred twenty-three completed the trial, 555 provided consent for Medicare, complete health related quality of life data for 535.
